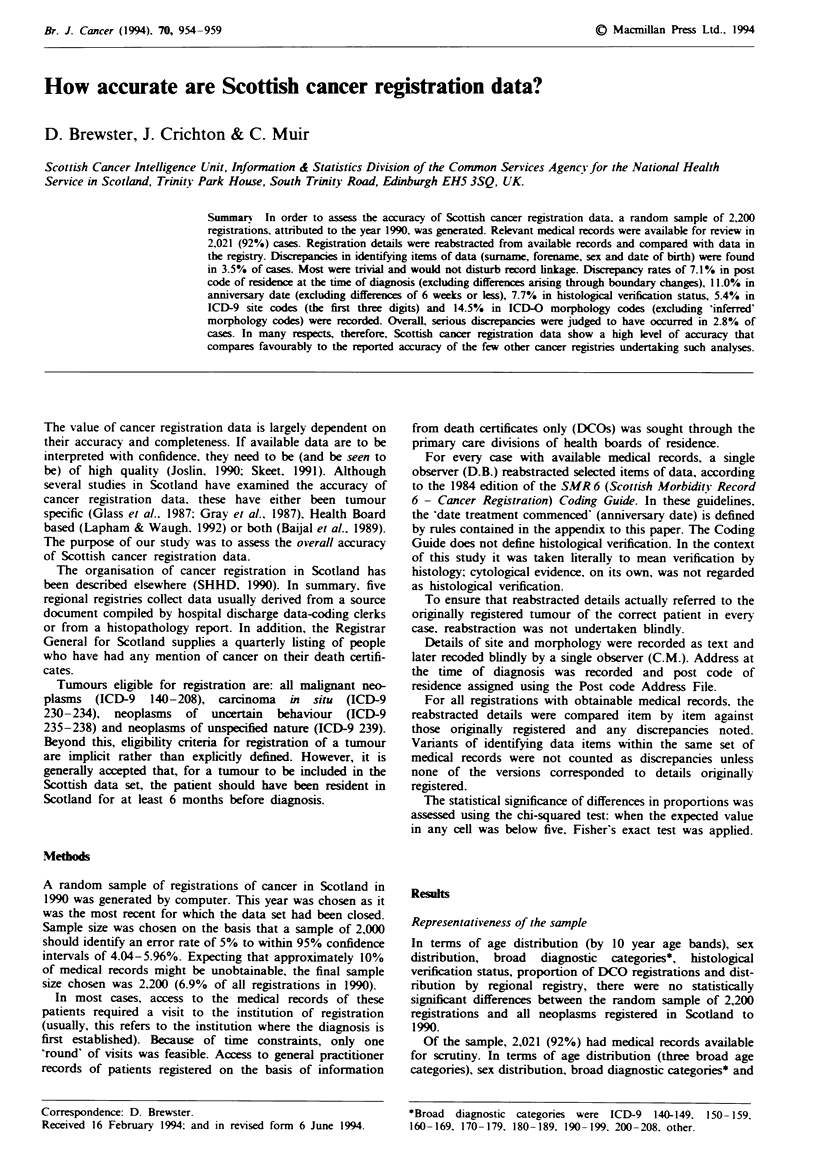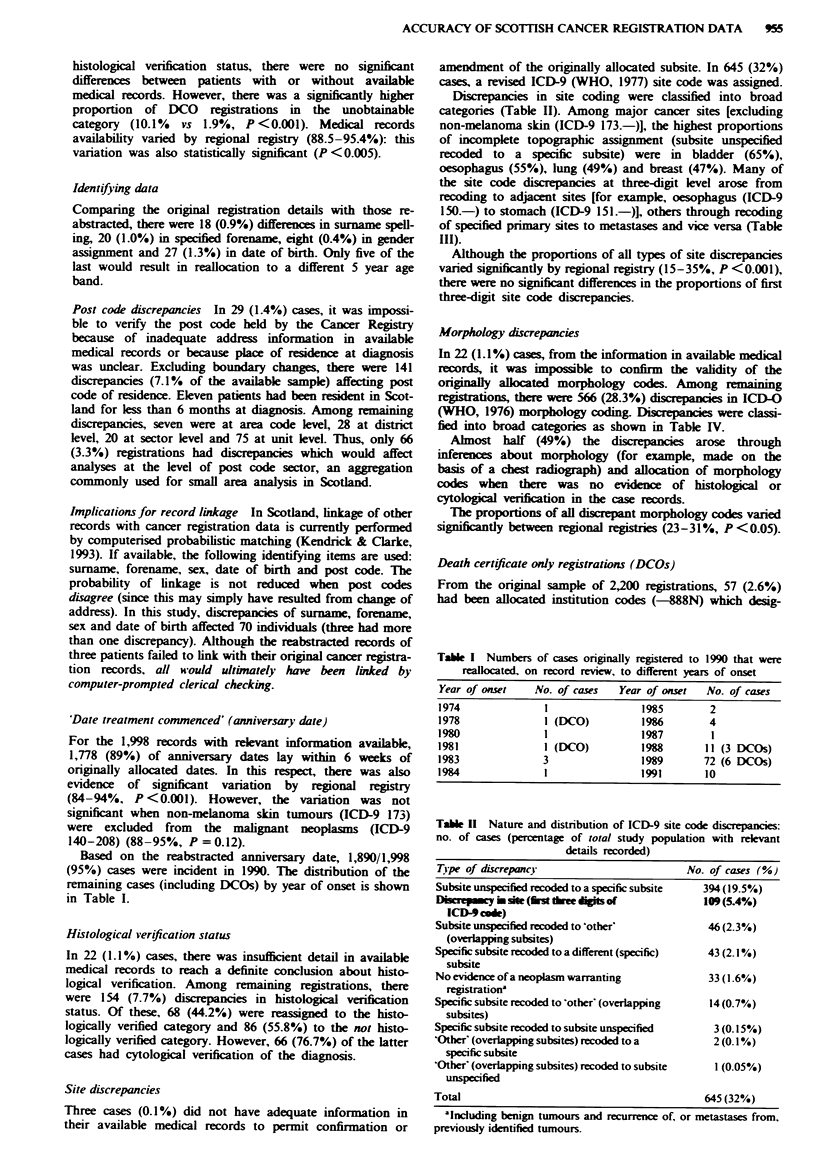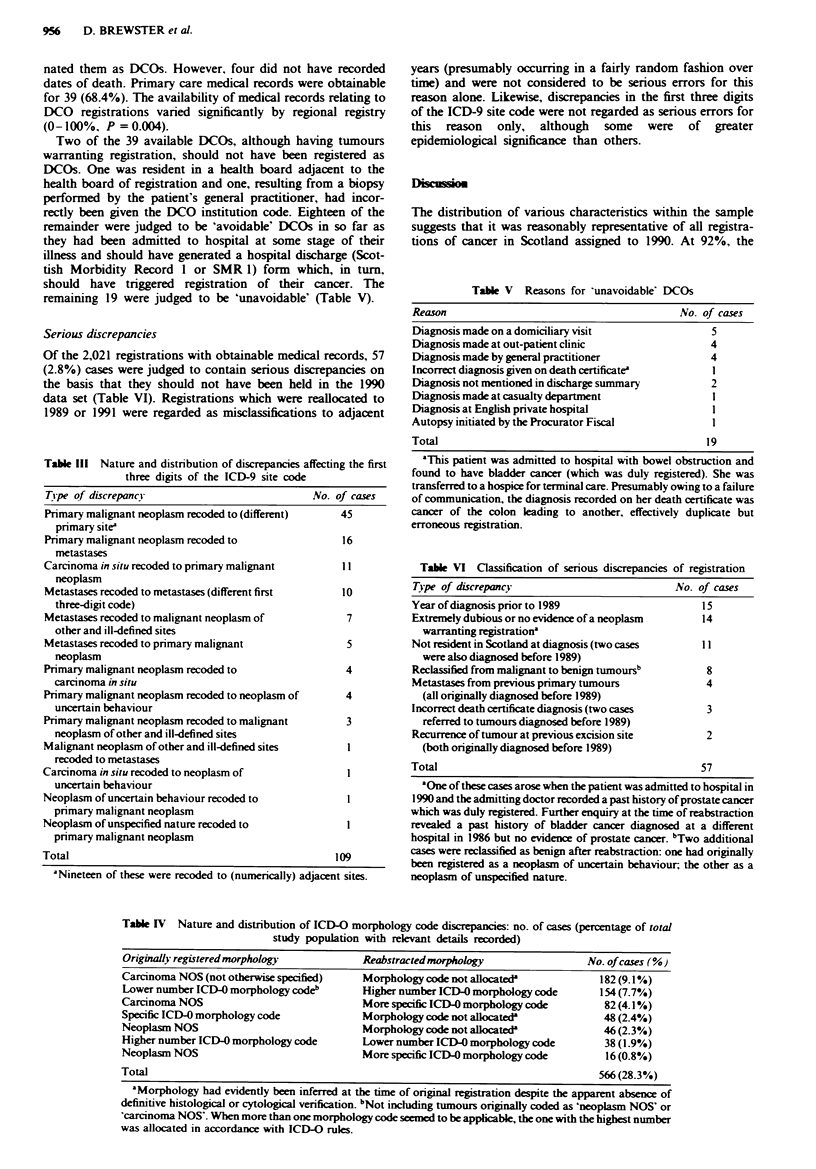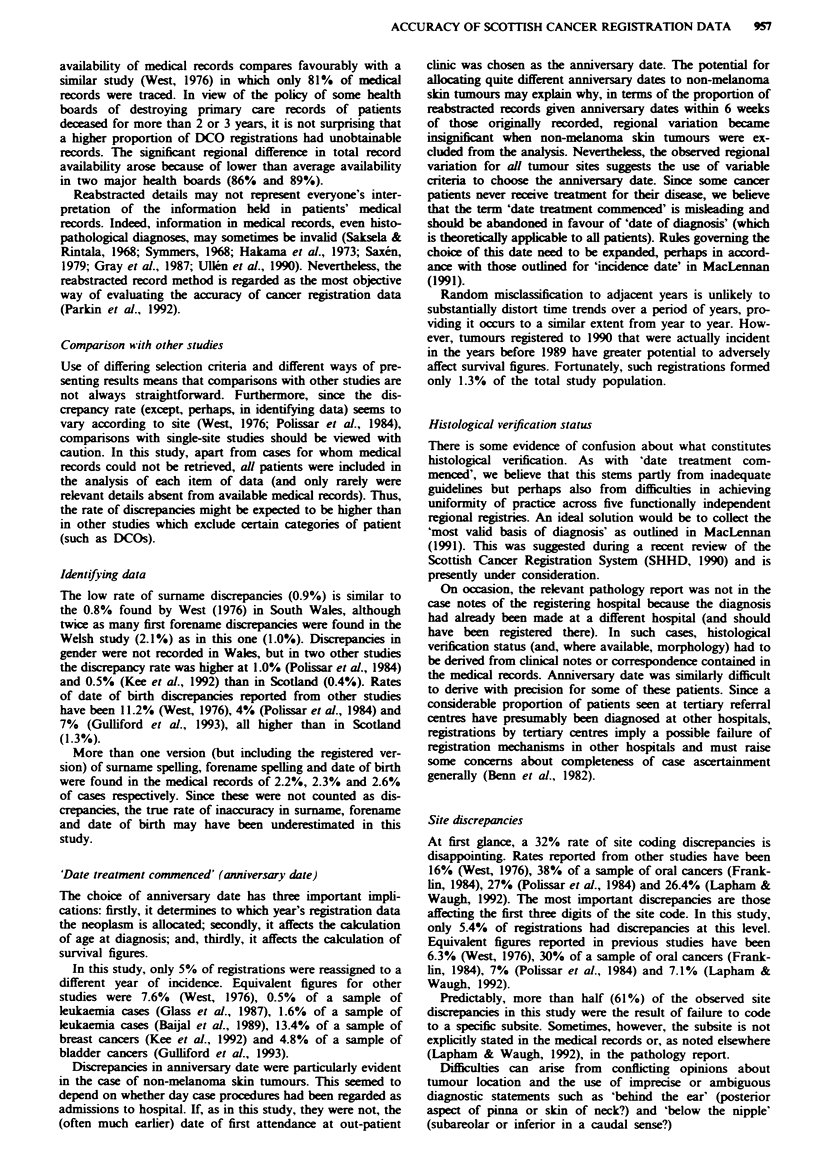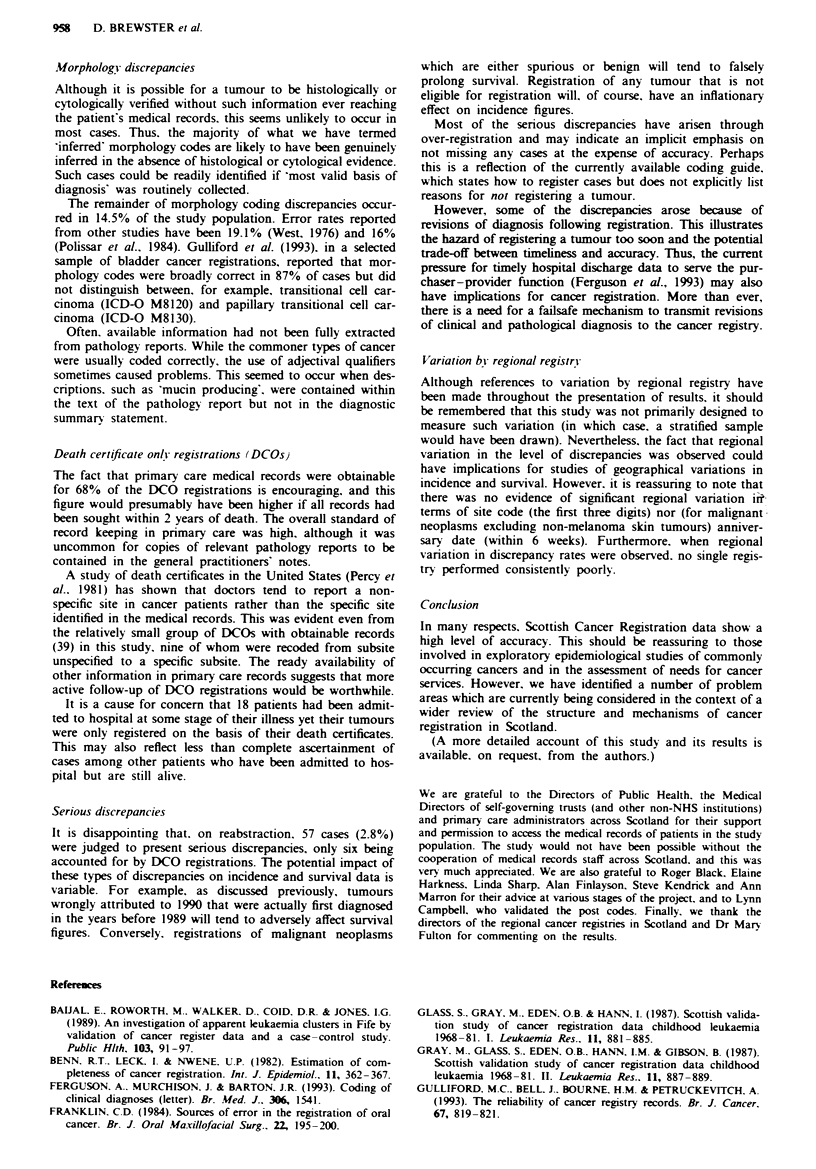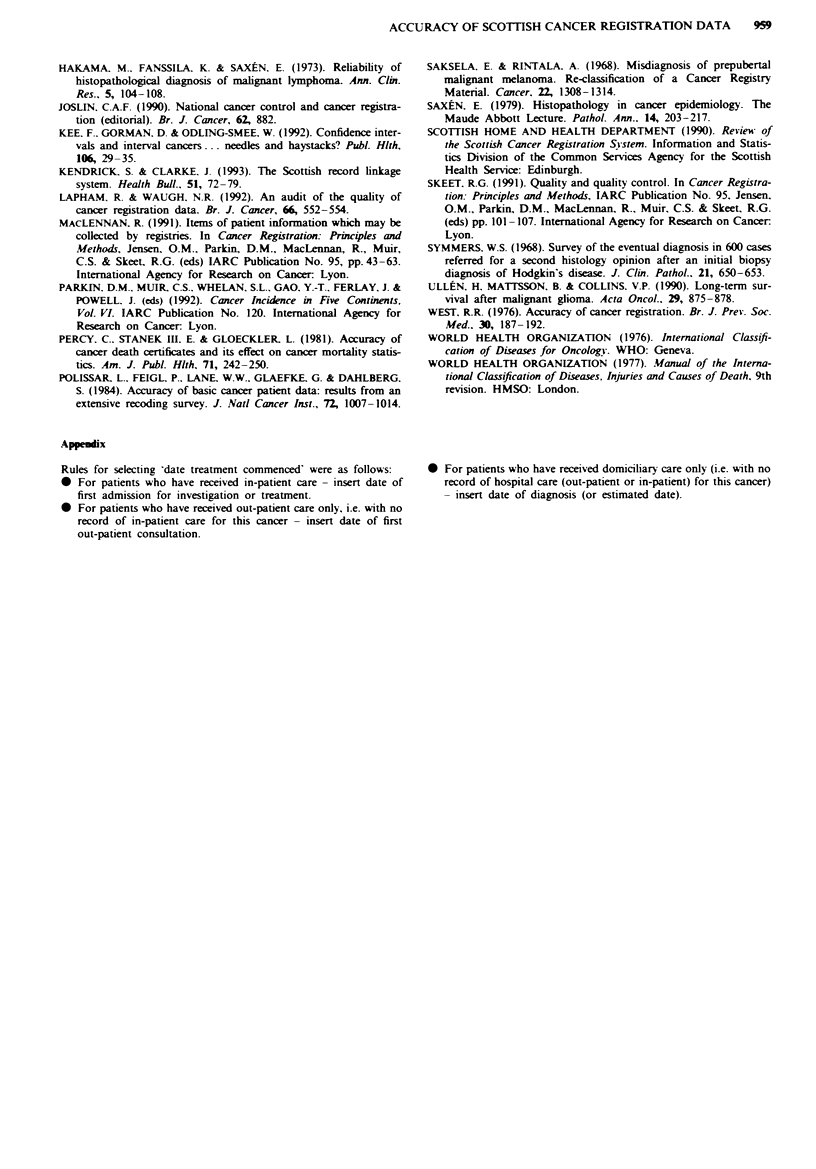# How accurate are Scottish cancer registration data?

**DOI:** 10.1038/bjc.1994.428

**Published:** 1994-11

**Authors:** D. Brewster, J. Crichton, C. Muir

**Affiliations:** Scottish Cancer Intelligence Unit, Information & Statistics Division of the Common Services Agency for the National Health Service in Scotland, Edinburgh, UK.

## Abstract

In order to assess the accuracy of Scottish cancer registration data, a random sample of 2,200 registrations, attributed to the year 1990, was generated. Relevant medical records were available for review in 2,021 (92%) cases. Registration details were reabstracted from available records and compared with data in the registry. Discrepancies in identifying items of data (surname, forename, sex and date of birth) were found in 3.5% of cases. Most were trivial and would not disturb record linkage. Discrepancy rates of 7.1% in post code of residence at the time of diagnosis (excluding differences arising through boundary changes), 11.0% in anniversary date (excluding differences of 6 weeks or less), 7.7% in histological verification status, 5.4% in ICD-9 site codes (the first three digits) and 14.5% in ICD-O morphology codes (excluding 'inferred' morphology codes) were recorded. Overall, serious discrepancies were judged to have occurred in 2.8% of cases. In many respects, therefore, Scottish cancer registration data show a high level of accuracy that compares favourably to the reported accuracy of the few other cancer registries undertaking such analyses.


*Br. J. Cancer* (1994), **70**, 954-959 © Macmillan Press Ltd., 1994

### How accurate are Scottish cancer registration data?

D. Brewster, J. Crichton & C. Muir

*Scottish Cancer Intelligence Unit, Information & Statistics Division of the Common Services Agency for the National Health Service in Scotland, Trinity Park House, South Trinity Road, Edinburgh EH5 3SQ, UK.*

**Summary** In order to assess the accuracy of Scottish cancer registration data, a random sample of 2,200 registrations, attributed to the year 1990, was generated. Relevant medical records were available for review in 2,021 (92%) cases. Registration details were reabstracted from available records and compared with data in the registry. Discrepancies in identifying items of data (surname, forename, sex and date of birth) were found in 3.5% of cases. Most were trivial and would not disturb record linkage. Discrepancy rates of 7.1% in post code of residence at the time of diagnosis (excluding differences arising through boundary changes), 11.0% in anniversary date (excluding differences of 6 weeks or less), 7.7% in histological verification status, 5.4% in ICD-9 site codes (the first three digits) and 14.5% in ICD-O morphology codes (excluding 'inferred' morphology codes) were recorded. Overall, serious discrepancies were judged to have occurred in 2.8% of cases. In many respects, therefore, Scottish cancer registration data show a high level of accuracy that compares favourably to the reported accuracy of the few other cancer registries undertaking such analyses.

The value of cancer registration data is largely dependent on their accuracy and completeness. If available data are to be interpreted with confidence, they need to be (and be *seen* to be) of high quality (Joslin, 1990; Skeet, 1991). Although several studies in Scotland have examined the accuracy of cancer registration data, these have either been tumour specific (Glass *et al.*, 1987; Gray *et al.*, 1987), Health Board based (Lapham & Waugh, 1992) or both (Baijal *et al.*, 1989). The purpose of our study was to assess the *overall* accuracy of Scottish cancer registration data.

The organisation of cancer registration in Scotland has been described elsewhere (SHHD, 1990). In summary, five regional registries collect data usually derived from a source document compiled by hospital discharge data-coding clerks or from a histopathology report. In addition, the Registrar General for Scotland supplies a quarterly listing of people who have had any mention of cancer on their death certificates.

Tumours eligible for registration are: all malignant neoplasms (ICD-9 140-208), carcinoma *in situ* (ICD-9 230-234), neoplasms of uncertain behaviour (ICD-9 235-238) and neoplasms of unspecified nature (ICD-9 239). Beyond this, eligibility criteria for registration of a tumour are implicit rather than explicitly defined. However, it is generally accepted that, for a tumour to be included in the Scottish data set, the patient should have been resident in Scotland for at least 6 months before diagnosis.

### Methods

A random sample of registrations of cancer in Scotland in 1990 was generated by computer. This year was chosen as it was the most recent for which the data set had been closed. Sample size was chosen on the basis that a sample of 2,000 should identify an error rate of 5% to within 95% confidence intervals of 4.04-5.96%. Expecting that approximately 10% of medical records might be unobtainable, the final sample size chosen was 2,200 (6.9% of all registrations in 1990).

In most cases, access to the medical records of these patients required a visit to the institution of registration (usually, this refers to the institution where the diagnosis is first established). Because of time constraints, only one 'round' of visits was feasible. Access to general practitioner records of patients registered on the basis of information from death certificates only (DCOs) was sought through the primary care divisions of health boards of residence.

For every case with available medical records, a single observer (D.B.) reabstracted selected items of data, according to the 1984 edition of the *SMR 6* (*Scottish Morbidity Record 6 - Cancer Registration*) *Coding Guide*. In these guidelines, the 'date treatment commenced' (anniversary date) is defined by rules contained in the appendix to this paper. The Coding Guide does not define histological verification. In the context of this study it was taken literally to mean verification by histology; cytological evidence, on its own, was not regarded as histological verification.

To ensure that reabstracted details actually referred to the originally registered tumour of the correct patient in every case, reabstraction was not undertaken blindly.

Details of site and morphology were recorded as text and later recoded blindly by a single observer (C.M.). Address at the time of diagnosis was recorded and post code of residence assigned using the Post code Address File.

For all registrations with obtainable medical records, the reabstracted details were compared item by item against those originally registered and any discrepancies noted. Variants of identifying data items within the same set of medical records were not counted as discrepancies unless none of the versions corresponded to details originally registered.

The statistical significance of differences in proportions was assessed using the chi-squared test: when the expected value in any cell was below five, Fisher's exact test was applied.

### Results

### *Representativeness of the sample*

In terms of age distribution (by 10 year age bands), sex distribution, broad diagnostic categories*, histological verification status, proportion of DCO registrations and distribution by regional registry, there were no statistically significant differences between the random sample of 2,200 registrations and all neoplasms registered in Scotland to 1990.

Of the sample, 2,021 (92%) had medical records available for scrutiny. In terms of age distribution (three broad age categories), sex distribution, broad diagnostic categories* and

Correspondence: D. Brewster.
Received 16 February 1994; and in revised form 6 June 1994.

*Broad diagnostic categories were ICD-9 140-149, 150-159, 160-169, 170-179, 180-189, 190-199, 200-208, other.

histological verification status, there were no significant differences between patients with or without available medical records. However, there was a significantly higher proportion of DCO registrations in the unobtainable category (10.1% *vs* 1.9%, $P < 0.001$). Medical records availability varied by regional registry (88.5–95.4%): this variation was also statistically significant ($P < 0.005$).

*Identifying data*

Comparing the original registration details with those re-abstracted, there were 18 (0.9%) differences in surname spelling, 20 (1.0%) in specified forename, eight (0.4%) in gender assignment and 27 (1.3%) in date of birth. Only five of the last would result in reallocation to a different 5 year age band.

*Post code discrepancies* In 29 (1.4%) cases, it was impossible to verify the post code held by the Cancer Registry because of inadequate address information in available medical records or because place of residence at diagnosis was unclear. Excluding boundary changes, there were 141 discrepancies (7.1% of the available sample) affecting post code of residence. Eleven patients had been resident in Scotland for less than 6 months at diagnosis. Among remaining discrepancies, seven were at area code level, 28 at district level, 20 at sector level and 75 at unit level. Thus, only 66 (3.3%) registrations had discrepancies which would affect analyses at the level of post code sector, an aggregation commonly used for small area analysis in Scotland.

*Implications for record linkage* In Scotland, linkage of other records with cancer registration data is currently performed by computerised probabilistic matching (Kendrick & Clarke, 1993). If available, the following identifying items are used: surname, forename, sex, date of birth and post code. The probability of linkage is not reduced when post codes *disagree* (since this may simply have resulted from change of address). In this study, discrepancies of surname, forename, sex and date of birth affected 70 individuals (three had more than one discrepancy). Although the reabstracted records of three patients failed to link with their original cancer registration records, *all would ultimately have been linked by computer-prompted clerical checking.*

*'Date treatment commenced' (anniversary date)*

For the 1,998 records with relevant information available, 1,778 (89%) of anniversary dates lay within 6 weeks of originally allocated dates. In this respect, there was also evidence of significant variation by regional registry (84–94%, $P < 0.001$). However, the variation was not significant when non-melanoma skin tumours (ICD-9 173) were excluded from the malignant neoplasms (ICD-9 140–208) (88–95%, $P = 0.12$).

Based on the reabstracted anniversary date, 1,890/1,998 (95%) cases were incident in 1990. The distribution of the remaining cases (including DCOs) by year of onset is shown in Table I.

*Histological verification status*

In 22 (1.1%) cases, there was insufficient detail in available medical records to reach a definite conclusion about histological verification. Among remaining registrations, there were 154 (7.7%) discrepancies in histological verification status. Of these, 68 (44.2%) were reassigned to the histologically verified category and 86 (55.8%) to the *not* histologically verified category. However, 66 (76.7%) of the latter cases had cytological verification of the diagnosis.

*Site discrepancies*

Three cases (0.1%) did not have adequate information in their available medical records to permit confirmation or amendment of the originally allocated subsite. In 645 (32%) cases, a revised ICD-9 (WHO, 1977) site code was assigned.

Discrepancies in site coding were classified into broad categories (Table II). Among major cancer sites [excluding non-melanoma skin (ICD-9 173.—)], the highest proportions of incomplete topographic assignment (subsite unspecified recoded to a specific subsite) were in bladder (65%), oesophagus (55%), lung (49%) and breast (47%). Many of the site code discrepancies at three-digit level arose from recoding to adjacent sites [for example, oesophagus (ICD-9 150.—) to stomach (ICD-9 151.—)], others through recoding of specified primary sites to metastases and vice versa (Table III).

Although the proportions of all types of site discrepancies varied significantly by regional registry (15–35%, $P < 0.001$), there were no significant differences in the proportions of first three-digit site code discrepancies.

*Morphology discrepancies*

In 22 (1.1%) cases, from the information in available medical records, it was impossible to confirm the validity of the originally allocated morphology codes. Among remaining registrations, there were 566 (28.3%) discrepancies in ICD-O (WHO, 1976) morphology coding. Discrepancies were classified into broad categories as shown in Table IV.

Almost half (49%) the discrepancies arose through inferences about morphology (for example, made on the basis of a chest radiograph) and allocation of morphology codes when there was no evidence of histological or cytological verification in the case records.

The proportions of all discrepant morphology codes varied significantly between regional registries (23–31%, $P < 0.05$).

*Death certificate only registrations (DCOs)*

From the original sample of 2,200 registrations, 57 (2.6%) had been allocated institution codes (—888N) which desig-

**Table I** Numbers of cases originally registered to 1990 that were reallocated, on record review, to different years of onset

| *Year of onset* | *No. of cases* | *Year of onset* | *No. of cases* |
|---|---|---|---|
| 1974 | 1 | 1985 | 2 |
| 1978 | 1 (DCO) | 1986 | 4 |
| 1980 | 1 | 1987 | 1 |
| 1981 | 1 (DCO) | 1988 | 11 (3 DCOs) |
| 1983 | 3 | 1989 | 72 (6 DCOs) |
| 1984 | 1 | 1991 | 10 |

**Table II** Nature and distribution of ICD-9 site code discrepancies: no. of cases (percentage of *total* study population with relevant details recorded)

| *Type of discrepancy* | *No. of cases (%)* |
|---|---|
| Subsite unspecified recoded to a specific subsite | 394 (19.5%) |
| **Discrepancy in site (first three digits of ICD-9 code)** | **109 (5.4%)** |
| Subsite unspecified recoded to 'other' (overlapping subsites) | 46 (2.3%) |
| Specific subsite recoded to a different (specific) subsite | 43 (2.1%) |
| No evidence of a neoplasm warranting registration[a] | 33 (1.6%) |
| Specific subsite recoded to 'other' (overlapping subsites) | 14 (0.7%) |
| Specific subsite recoded to subsite unspecified | 3 (0.15%) |
| 'Other' (overlapping subsites) recoded to a specific subsite | 2 (0.1%) |
| 'Other' (overlapping subsites) recoded to subsite unspecified | 1 (0.05%) |
| Total | 645 (32%) |

[a]Including benign tumours and recurrence of, or metastases from, previously identified tumours.

nated them as DCOs. However, four did not have recorded dates of death. Primary care medical records were obtainable for 39 (68.4%). The availability of medical records relating to DCO registrations varied significantly by regional registry (0–100%, $P = 0.004$).

Two of the 39 available DCOs, although having tumours warranting registration, should not have been registered as DCOs. One was resident in a health board adjacent to the health board of registration and one, resulting from a biopsy performed by the patient's general practitioner, had incorrectly been given the DCO institution code. Eighteen of the remainder were judged to be 'avoidable' DCOs in so far as they had been admitted to hospital at some stage of their illness and should have generated a hospital discharge (Scottish Morbidity Record 1 or SMR 1) form which, in turn, should have triggered registration of their cancer. The remaining 19 were judged to be 'unavoidable' (Table V).

*Serious discrepancies*

Of the 2,021 registrations with obtainable medical records, 57 (2.8%) cases were judged to contain serious discrepancies on the basis that they should not have been held in the 1990 data set (Table VI). Registrations which were reallocated to 1989 or 1991 were regarded as misclassifications to adjacent years (presumably occurring in a fairly random fashion over time) and were not considered to be serious errors for this reason alone. Likewise, discrepancies in the first three digits of the ICD-9 site code were not regarded as serious errors for this reason only, although some were of greater epidemiological significance than others.

### Discussion

The distribution of various characteristics within the sample suggests that it was reasonably representative of all registrations of cancer in Scotland assigned to 1990. At 92%, the

**Table III** Nature and distribution of discrepancies affecting the first three digits of the ICD-9 site code

| *Type of discrepancy* | *No. of cases* |
|---|---|
| Primary malignant neoplasm recoded to (different) primary site[a] | 45 |
| Primary malignant neoplasm recoded to metastases | 16 |
| Carcinoma *in situ* recoded to primary malignant neoplasm | 11 |
| Metastases recoded to metastases (different first three-digit code) | 10 |
| Metastases recoded to malignant neoplasm of other and ill-defined sites | 7 |
| Metastases recoded to primary malignant neoplasm | 5 |
| Primary malignant neoplasm recoded to carcinoma *in situ* | 4 |
| Primary malignant neoplasm recoded to neoplasm of uncertain behaviour | 4 |
| Primary malignant neoplasm recoded to malignant neoplasm of other and ill-defined sites | 3 |
| Malignant neoplasm of other and ill-defined sites recoded to metastases | 1 |
| Carcinoma *in situ* recoded to neoplasm of uncertain behaviour | 1 |
| Neoplasm of uncertain behaviour recoded to primary malignant neoplasm | 1 |
| Neoplasm of unspecified nature recoded to primary malignant neoplasm | 1 |
| Total | 109 |

[a]Nineteen of these were recoded to (numerically) adjacent sites.

**Table V** Reasons for 'unavoidable' DCOs

| *Reason* | *No. of cases* |
|---|---|
| Diagnosis made on a domiciliary visit | 5 |
| Diagnosis made at out-patient clinic | 4 |
| Diagnosis made by general practitioner | 4 |
| Incorrect diagnosis given on death certificate[a] | 1 |
| Diagnosis not mentioned in discharge summary | 2 |
| Diagnosis made at casualty department | 1 |
| Diagnosis at English private hospital | 1 |
| Autopsy initiated by the Procurator Fiscal | 1 |
| Total | 19 |

[a]This patient was admitted to hospital with bowel obstruction and found to have bladder cancer (which was duly registered). She was transferred to a hospice for terminal care. Presumably owing to a failure of communication, the diagnosis recorded on her death certificate was cancer of the colon leading to another, effectively duplicate but erroneous registration.

**Table VI** Classification of serious discrepancies of registration

| *Type of discrepancy* | *No. of cases* |
|---|---|
| Year of diagnosis prior to 1989 | 15 |
| Extremely dubious or no evidence of a neoplasm warranting registration[a] | 14 |
| Not resident in Scotland at diagnosis (two cases were also diagnosed before 1989) | 11 |
| Reclassified from malignant to benign tumours[b] | 8 |
| Metastases from previous primary tumours (all originally diagnosed before 1989) | 4 |
| Incorrect death certificate diagnosis (two cases referred to tumours diagnosed before 1989) | 3 |
| Recurrence of tumour at previous excision site (both originally diagnosed before 1989) | 2 |
| Total | 57 |

[a]One of these cases arose when the patient was admitted to hospital in 1990 and the admitting doctor recorded a past history of prostate cancer which was duly registered. Further enquiry at the time of reabstraction revealed a past history of bladder cancer diagnosed at a different hospital in 1986 but no evidence of prostate cancer. [b]Two additional cases were reclassified as benign after reabstraction: one had originally been registered as a neoplasm of uncertain behaviour; the other as a neoplasm of unspecified nature.

**Table IV** Nature and distribution of ICD-O morphology code discrepancies: no. of cases (percentage of *total* study population with relevant details recorded)

| *Originally registered morphology* | *Reabstracted morphology* | *No. of cases (%)* |
|---|---|---|
| Carcinoma NOS (not otherwise specified) | Morphology code not allocated[a] | 182 (9.1%) |
| Lower number ICD-0 morphology code[b] | Higher number ICD-0 morphology code | 154 (7.7%) |
| Carcinoma NOS | More specific ICD-0 morphology code | 82 (4.1%) |
| Specific ICD-0 morphology code | Morphology code not allocated[a] | 48 (2.4%) |
| Neoplasm NOS | Morphology code not allocated[a] | 46 (2.3%) |
| Higher number ICD-0 morphology code | Lower number ICD-0 morphology code | 38 (1.9%) |
| Neoplasm NOS | More specific ICD-0 morphology code | 16 (0.8%) |
| Total | | 566 (28.3%) |

[a]Morphology had evidently been inferred at the time of original registration despite the apparent absence of definitive histological or cytological verification. [b]Not including tumours originally coded as 'neoplasm NOS' or 'carcinoma NOS'. When more than one morphology code seemed to be applicable, the one with the highest number was allocated in accordance with ICD-O rules.

availability of medical records compares favourably with a similar study (West, 1976) in which only 81% of medical records were traced. In view of the policy of some health boards of destroying primary care records of patients deceased for more than 2 or 3 years, it is not surprising that a higher proportion of DCO registrations had unobtainable records. The significant regional difference in total record availability arose because of lower than average availability in two major health boards (86% and 89%).

Reabstracted details may not represent everyone's interpretation of the information held in patients' medical records. Indeed, information in medical records, even histopathological diagnoses, may sometimes be invalid (Saksela & Rintala, 1968; Symmers, 1968; Hakama *et al.*, 1973; Saxén, 1979; Gray *et al.*, 1987; Ullén *et al.*, 1990). Nevertheless, the reabstracted record method is regarded as the most objective way of evaluating the accuracy of cancer registration data (Parkin *et al.*, 1992).

### *Comparison with other studies*

Use of differing selection criteria and different ways of presenting results means that comparisons with other studies are not always straightforward. Furthermore, since the discrepancy rate (except, perhaps, in identifying data) seems to vary according to site (West, 1976; Polissar *et al.*, 1984), comparisons with single-site studies should be viewed with caution. In this study, apart from cases for whom medical records could not be retrieved, *all* patients were included in the analysis of each item of data (and only rarely were relevant details absent from available medical records). Thus, the rate of discrepancies might be expected to be higher than in other studies which exclude certain categories of patient (such as DCOs).

### *Identifying data*

The low rate of surname discrepancies (0.9%) is similar to the 0.8% found by West (1976) in South Wales, although twice as many first forename discrepancies were found in the Welsh study (2.1%) as in this one (1.0%). Discrepancies in gender were not recorded in Wales, but in two other studies the discrepancy rate was higher at 1.0% (Polissar *et al.*, 1984) and 0.5% (Kee *et al.*, 1992) than in Scotland (0.4%). Rates of date of birth discrepancies reported from other studies have been 11.2% (West, 1976), 4% (Polissar *et al.*, 1984) and 7% (Gulliford *et al.*, 1993), all higher than in Scotland (1.3%).

More than one version (but including the registered version) of surname spelling, forename spelling and date of birth were found in the medical records of 2.2%, 2.3% and 2.6% of cases respectively. Since these were not counted as discrepancies, the true rate of inaccuracy in surname, forename and date of birth may have been underestimated in this study.

### *'Date treatment commenced' (anniversary date)*

The choice of anniversary date has three important implications: firstly, it determines to which year's registration data the neoplasm is allocated; secondly, it affects the calculation of age at diagnosis; and, thirdly, it affects the calculation of survival figures.

In this study, only 5% of registrations were reassigned to a different year of incidence. Equivalent figures for other studies were 7.6% (West, 1976), 0.5% of a sample of leukaemia cases (Glass *et al.*, 1987), 1.6% of a sample of leukaemia cases (Baijal *et al.*, 1989), 13.4% of a sample of breast cancers (Kee *et al.*, 1992) and 4.8% of a sample of bladder cancers (Gulliford *et al.*, 1993).

Discrepancies in anniversary date were particularly evident in the case of non-melanoma skin tumours. This seemed to depend on whether day case procedures had been regarded as admissions to hospital. If, as in this study, they were not, the (often much earlier) date of first attendance at out-patient clinic was chosen as the anniversary date. The potential for allocating quite different anniversary dates to non-melanoma skin tumours may explain why, in terms of the proportion of reabstracted records given anniversary dates within 6 weeks of those originally recorded, regional variation became insignificant when non-melanoma skin tumours were excluded from the analysis. Nevertheless, the observed regional variation for *all* tumour sites suggests the use of variable criteria to choose the anniversary date. Since some cancer patients never receive treatment for their disease, we believe that the term 'date treatment commenced' is misleading and should be abandoned in favour of 'date of diagnosis' (which is theoretically applicable to all patients). Rules governing the choice of this date need to be expanded, perhaps in accordance with those outlined for 'incidence date' in MacLennan (1991).

Random misclassification to adjacent years is unlikely to substantially distort time trends over a period of years, providing it occurs to a similar extent from year to year. However, tumours registered to 1990 that were actually incident in the years before 1989 have greater potential to adversely affect survival figures. Fortunately, such registrations formed only 1.3% of the total study population.

### *Histological verification status*

There is some evidence of confusion about what constitutes histological verification. As with 'date treatment commenced', we believe that this stems partly from inadequate guidelines but perhaps also from difficulties in achieving uniformity of practice across five functionally independent regional registries. An ideal solution would be to collect the 'most valid basis of diagnosis' as outlined in MacLennan (1991). This was suggested during a recent review of the Scottish Cancer Registration System (SHHD, 1990) and is presently under consideration.

On occasion, the relevant pathology report was not in the case notes of the registering hospital because the diagnosis had already been made at a different hospital (and should have been registered there). In such cases, histological verification status (and, where available, morphology) had to be derived from clinical notes or correspondence contained in the medical records. Anniversary date was similarly difficult to derive with precision for some of these patients. Since a considerable proportion of patients seen at tertiary referral centres have presumably been diagnosed at other hospitals, registrations by tertiary centres imply a possible failure of registration mechanisms in other hospitals and must raise some concerns about completeness of case ascertainment generally (Benn *et al.*, 1982).

### *Site discrepancies*

At first glance, a 32% rate of site coding discrepancies is disappointing. Rates reported from other studies have been 16% (West, 1976), 38% of a sample of oral cancers (Franklin, 1984), 27% (Polissar *et al.*, 1984) and 26.4% (Lapham & Waugh, 1992). The most important discrepancies are those affecting the first three digits of the site code. In this study, only 5.4% of registrations had discrepancies at this level. Equivalent figures reported in previous studies have been 6.3% (West, 1976), 30% of a sample of oral cancers (Franklin, 1984), 7% (Polissar *et al.*, 1984) and 7.1% (Lapham & Waugh, 1992).

Predictably, more than half (61%) of the observed site discrepancies in this study were the result of failure to code to a specific subsite. Sometimes, however, the subsite is not explicitly stated in the medical records or, as noted elsewhere (Lapham & Waugh, 1992), in the pathology report.

Difficulties can arise from conflicting opinions about tumour location and the use of imprecise or ambiguous diagnostic statements such as 'behind the ear' (posterior aspect of pinna or skin of neck?) and 'below the nipple' (subareolar or inferior in a caudal sense?)

*Morphology discrepancies*

Although it is possible for a tumour to be histologically or cytologically verified without such information ever reaching the patient's medical records, this seems unlikely to occur in most cases. Thus, the majority of what we have termed 'inferred' morphology codes are likely to have been genuinely inferred in the absence of histological or cytological evidence. Such cases could be readily identified if 'most valid basis of diagnosis' was routinely collected.

The remainder of morphology coding discrepancies occurred in 14.5% of the study population. Error rates reported from other studies have been 19.1% (West, 1976) and 16% (Polissar *et al.*, 1984). Gulliford *et al.* (1993), in a selected sample of bladder cancer registrations, reported that morphology codes were broadly correct in 87% of cases but did not distinguish between, for example, transitional cell carcinoma (ICD-O M8120) and papillary transitional cell carcinoma (ICD-O M8130).

Often, available information had not been fully extracted from pathology reports. While the commoner types of cancer were usually coded correctly, the use of adjectival qualifiers sometimes caused problems. This seemed to occur when descriptions, such as 'mucin producing', were contained within the text of the pathology report but not in the diagnostic summary statement.

*Death certificate only registrations (DCOs)*

The fact that primary care medical records were obtainable for 68% of the DCO registrations is encouraging, and this figure would presumably have been higher if all records had been sought within 2 years of death. The overall standard of record keeping in primary care was high, although it was uncommon for copies of relevant pathology reports to be contained in the general practitioners' notes.

A study of death certificates in the United States (Percy *et al.*, 1981) has shown that doctors tend to report a non-specific site in cancer patients rather than the specific site identified in the medical records. This was evident even from the relatively small group of DCOs with obtainable records (39) in this study, nine of whom were recoded from subsite unspecified to a specific subsite. The ready availability of other information in primary care records suggests that more active follow-up of DCO registrations would be worthwhile.

It is a cause for concern that 18 patients had been admitted to hospital at some stage of their illness yet their tumours were only registered on the basis of their death certificates. This may also reflect less than complete ascertainment of cases among other patients who have been admitted to hospital but are still alive.

*Serious discrepancies*

It is disappointing that, on reabstraction, 57 cases (2.8%) were judged to present serious discrepancies, only six being accounted for by DCO registrations. The potential impact of these types of discrepancies on incidence and survival data is variable. For example, as discussed previously, tumours wrongly attributed to 1990 that were actually first diagnosed in the years before 1989 will tend to adversely affect survival figures. Conversely, registrations of malignant neoplasms which are either spurious or benign will tend to falsely prolong survival. Registration of any tumour that is not eligible for registration will, of course, have an inflationary effect on incidence figures.

Most of the serious discrepancies have arisen through over-registration and may indicate an implicit emphasis on not missing any cases at the expense of accuracy. Perhaps this is a reflection of the currently available coding guide, which states how to register cases but does not explicitly list reasons for *not* registering a tumour.

However, some of the discrepancies arose because of revisions of diagnosis following registration. This illustrates the hazard of registering a tumour too soon and the potential trade-off between timeliness and accuracy. Thus, the current pressure for timely hospital discharge data to serve the purchaser–provider function (Ferguson *et al.*, 1993) may also have implications for cancer registration. More than ever, there is a need for a failsafe mechanism to transmit revisions of clinical and pathological diagnosis to the cancer registry.

*Variation by regional registry*

Although references to variation by regional registry have been made throughout the presentation of results, it should be remembered that this study was not primarily designed to measure such variation (in which case, a stratified sample would have been drawn). Nevertheless, the fact that regional variation in the level of discrepancies was observed could have implications for studies of geographical variations in incidence and survival. However, it is reassuring to note that there was no evidence of significant regional variation in terms of site code (the first three digits) nor (for malignant neoplasms excluding non-melanoma skin tumours) anniversary date (within 6 weeks). Furthermore, when regional variation in discrepancy rates were observed, no single registry performed consistently poorly.

*Conclusion*

In many respects, Scottish Cancer Registration data show a high level of accuracy. This should be reassuring to those involved in exploratory epidemiological studies of commonly occurring cancers and in the assessment of needs for cancer services. However, we have identified a number of problem areas which are currently being considered in the context of a wider review of the structure and mechanisms of cancer registration in Scotland.

(A more detailed account of this study and its results is available, on request, from the authors.)

We are grateful to the Directors of Public Health, the Medical Directors of self-governing trusts (and other non-NHS institutions) and primary care administrators across Scotland for their support and permission to access the medical records of patients in the study population. The study would not have been possible without the cooperation of medical records staff across Scotland, and this was very much appreciated. We are also grateful to Roger Black, Elaine Harkness, Linda Sharp, Alan Finlayson, Steve Kendrick and Ann Marron for their advice at various stages of the project, and to Lynn Campbell, who validated the post codes. Finally, we thank the directors of the regional cancer registries in Scotland and Dr Mary Fulton for commenting on the results.

**References**

BAIJAL, E., ROWORTH, M., WALKER, D., COID, D.R. & JONES, I.G. (1989). An investigation of apparent leukaemia clusters in Fife by validation of cancer register data and a case–control study. *Public Hlth*, **103**, 91–97.

BENN, R.T., LECK, I. & NWENE, U.P. (1982). Estimation of completeness of cancer registration. *Int. J. Epidemiol.*, **11**, 362–367.

FERGUSON, A., MURCHISON, J. & BARTON, J.R. (1993). Coding of clinical diagnoses (letter). *Br. Med. J.*, **306**, 1541.

FRANKLIN, C.D. (1984). Sources of error in the registration of oral cancer. *Br. J. Oral Maxillofacial Surg.*, **22**, 195–200.

GLASS, S., GRAY, M., EDEN, O.B. & HANN, I. (1987). Scottish validation study of cancer registration data childhood leukaemia 1968–81. I. *Leukaemia Res.*, **11**, 881–885.

GRAY, M., GLASS, S., EDEN, O.B., HANN, I.M. & GIBSON, B. (1987). Scottish validation study of cancer registration data childhood leukaemia 1968–81. II. *Leukaemia Res.*, **11**, 887–889.

GULLIFORD, M.C., BELL, J., BOURNE, H.M. & PETRUCKEVITCH, A. (1993). The reliability of cancer registry records. *Br. J. Cancer*, **67**, 819–821.

HAKAMA, M., FANSSILA, K. & SAXÉN, E. (1973). Reliability of histopathological diagnosis of malignant lymphoma. *Ann. Clin. Res.*, **5**, 104-108.

JOSLIN, C.A.F. (1990). National cancer control and cancer registration (editorial). *Br. J. Cancer*, **62**, 882.

KEE, F., GORMAN, D. & ODLING-SMEE, W. (1992). Confidence intervals and interval cancers . . . needles and haystacks? *Publ. Hlth*, **106**, 29-35.

KENDRICK, S. & CLARKE, J. (1993). The Scottish record linkage system. *Health Bull.*, **51**, 72-79.

LAPHAM, R. & WAUGH, N.R. (1992). An audit of the quality of cancer registration data. *Br. J. Cancer*, **66**, 552-554.

MACLENNAN, R. (1991). Items of patient information which may be collected by registries. In *Cancer Registration: Principles and Methods*, Jensen, O.M., Parkin, D.M., MacLennan, R., Muir, C.S. & Skeet, R.G. (eds) IARC Publication No. 95, pp. 43-63. International Agency for Research on Cancer: Lyon.

PARKIN, D.M., MUIR, C.S., WHELAN, S.L., GAO, Y.-T., FERLAY, J. & POWELL, J. (eds) (1992). *Cancer Incidence in Five Continents, Vol. VI*. IARC Publication No. 120. International Agency for Research on Cancer: Lyon.

PERCY, C., STANEK III, E. & GLOECKLER, L. (1981). Accuracy of cancer death certificates and its effect on cancer mortality statistics. *Am. J. Publ. Hlth*, **71**, 242-250.

POLISSAR, L., FEIGL, P., LANE, W.W., GLAEFKE, G. & DAHLBERG, S. (1984). Accuracy of basic cancer patient data: results from an extensive recoding survey. *J. Natl Cancer Inst.*, **72**, 1007-1014.

SAKSELA, E. & RINTALA, A. (1968). Misdiagnosis of prepubertal malignant melanoma. Re-classification of a Cancer Registry Material. *Cancer*, **22**, 1308-1314.

SAXÉN, E. (1979). Histopathology in cancer epidemiology. The Maude Abbott Lecture. *Pathol. Ann.*, **14**, 203-217.

SCOTTISH HOME AND HEALTH DEPARTMENT (1990). *Review of the Scottish Cancer Registration System*. Information and Statistics Division of the Common Services Agency for the Scottish Health Service: Edinburgh.

SKEET, R.G. (1991). Quality and quality control. In *Cancer Registration: Principles and Methods*, IARC Publication No. 95, Jensen, O.M., Parkin, D.M., MacLennan, R., Muir, C.S. & Skeet, R.G. (eds) pp. 101-107. International Agency for Research on Cancer: Lyon.

SYMMERS, W.S. (1968). Survey of the eventual diagnosis in 600 cases referred for a second histology opinion after an initial biopsy diagnosis of Hodgkin's disease. *J. Clin. Pathol.*, **21**, 650-653.

ULLÉN, H, MATTSSON, B. & COLLINS, V.P. (1990). Long-term survival after malignant glioma. *Acta Oncol.*, **29**, 875-878.

WEST, R.R. (1976). Accuracy of cancer registration. *Br. J. Prev. Soc. Med.*, **30**, 187-192.

WORLD HEALTH ORGANIZATION (1976). *International Classification of Diseases for Oncology*. WHO: Geneva.

WORLD HEALTH ORGANIZATION (1977). *Manual of the International Classification of Diseases, Injuries and Causes of Death*, 9th revision. HMSO: London.

**Appendix**

Rules for selecting 'date treatment commenced' were as follows:

- For patients who have received in-patient care – insert date of first admission for investigation or treatment.
- For patients who have received out-patient care only, i.e. with no record of in-patient care for this cancer – insert date of first out-patient consultation.
- For patients who have received domiciliary care only (i.e. with no record of hospital care (out-patient or in-patient) for this cancer) – insert date of diagnosis (or estimated date).